# Antioxidant Properties of Water-Soluble Gum from Flaxseed Hulls

**DOI:** 10.3390/antiox5030026

**Published:** 2016-08-02

**Authors:** Fatma Bouaziz, Mohamed Koubaa, Francisco J. Barba, Shahin Roohinejad, Semia Ellouz Chaabouni

**Affiliations:** 1 Enzyme Bioconversion Unit (UR13ES74), National School of Engineering, Sfax University, Sfax 3030, Tunisia; fatma.bouaziz22@yahoo.fr (F.B.); semia.chaabouni@enis.rnu.tn (S.E.C.); 2 Département de Génie des Procédés Industriels, Laboratoire Transformations Intégrées de la Matière Renouvelable (UTC/ESCOM, EA 4297 TIMR), Université de Technologie de Compiègne, Compiègne Cedex 60203, France; m.koubaa@escom.fr; 3 Faculty of Pharmacy, Universitat de València, Avda, Vicent Andrés Estellés, s/n 46100 Burjassot, València 46940, Spain; 4 Burn and Wound Healing Research Center, Division of Food and Nutrition, Shiraz University of Medical Sciences, Shiraz 7198754361, Iran; falcon.roh@gmail.com; 5 Common Service Unit of Bioreactor Coupled with an Ultrafilter, National School of Engineering, Sfax University, Sfax 3030, Tunisia

**Keywords:** flax hull, flaxseed gum, antioxidant properties

## Abstract

Soluble flaxseed gum (SFG) was extracted from flax (*Linum usitatissimum*) hulls using hot water, and its functional groups and antioxidant properties were investigated using infrared spectroscopy and different antioxidant assays (2,2-diphenyl-1-picrylhydrazyl (DPPH), 2,2′-azino-bis(3-ethylbenzothiazoline-6-sulphonic acid (ABTS), reducing power capacity, and β-carotene bleaching inhibition assay), respectively. The antioxidant capacity of SFG showed interesting DPPH radical-scavenging capacity (IC_50_ SFG = 2.5 mg·mL^−1^), strong ABTS radical scavenging activity (% inhibition ABTS = 75.6% ± 2.6% at 40 mg·mL^−1^), high reducing power capacity (RP_SFG_ = 5 mg·mL^−1^), and potent β-carotene bleaching inhibition activity (IC_50_ SFG = 10 mg·mL^−1^). All of the obtained results demonstrate the promising potential use of SFG in numerous industrial applications, and a way to valorize flaxseed hulls.

## 1. Introduction

Flax (*Linum usitatissimum* L.) was first cultivated as a fiber crop; nonetheless, its value and importance as an oil source has been interestingly increased [1]. From a health point of view, agri-food and pharmaceutical industries have shown a growing interest in flax seeds due to their richness in nutritionally valuable compounds, such as lignans (i.e., secoisolariciresinol diglucoside, α-linolenic acid, and soluble flaxseed gum (SFG) [2,3]. SFG, called also mucilage, is mainly present in the outermost layer of the seed hull [4], and can be released by soaking in water [5]. Previous works showed that a better extraction of SFG could be performed during 3 h at a temperature ranging from 85 to 95 °C, and a pH ranging from 6.5 to 7.0 [6]. The monosaccharide composition of SFG showed the presence of fucose, rhamnose, arabinose, galactose, glucose, and xylose, with the respective percentages of 7.0 ± 0.2, 16.5 ± 0.6, 12.7 ± 0.1, 22.4 ± 1.0, 2.7 ± 0.1, and 38.6 ± 1.2 [5]. SFG exhibits interesting features that allow it to be used in numerous industrial applications. For instance, its low viscosity (shear rate ranging from 10 to 1000 s^−1^) compared to other gums, such as locust bean gum, guar gum, and xanthan gum [7] gives it the promising fortification properties of fibers in food products [5]. Despite the numerous studies describing the health-related benefits of flax seeds and their food applications [8,9,10,11,12], little interest has been reported on the role of SFG in promoting human health [13,14]. Fractionating flax seed to kernel and hull—representing 63% and 37% of the total seed weight [15], respectively—is of great importance, as it allows an oil-rich fraction (kernel) and a gum-rich fraction (hull) to be obtained, thus reducing the downstream processing cost and increasing the profitability. Numerous studies have shown interest in valorizing flaxseed hulls by extracting the residual oil [16], proteins and polyphenols [17,18], as well as SFG, with some potential applications [19,20,21,22,23,24]. Investigating other health-related benefits of SFG—such as its antioxidant potential, which has not been described before—is interesting, as it may lead to the replacement of some synthetic antioxidants, which are more and more restricted by legislation [25]. In fact, water-soluble polysaccharides extracted from plant materials and by-products may exhibit antioxidant activities, such as those extracted from red prickly pear peels [26], almond gum [27], garlic straw [28], almond and pistachio juice processing by-products [29], and many other examples. In this line, this work aims at extracting SFG from hulls and evaluating its antioxidant properties.

## 2. Materials and Methods

### 2.1. Chemicals

ABTS (2,2′-azino-bis(3-ethylbenzothiazoline-6-sulphonic acid), DPPH (2,2-diphenyl-1- picrylhydrazyl), BHA (butylated hydroxyanisole), Trolox (6-hydroxy-2,5,7,8-tetramethylchroman- 2-carboxylic acid), sodium tetraborate, sodium phosphate, ferric chloride, petroleum ether, and trichloroacetic acid were purchased from Sigma-Aldrich (Darmstadt, Germany). Ethanol and sulfuric acid were purchased from Scharlab (Barcelona, Spain). Ammonium molybdate was obtained from NenTech Ltd. (London, UK), and potassium ferricyanide was obtained from Loba Chemie (Mumbai, India). Commercial gum arabic, purchased from a local market (Sfax, Tunisia), was the hydrocolloid used as reference.

### 2.2. Plant Material

**Figure 1:**
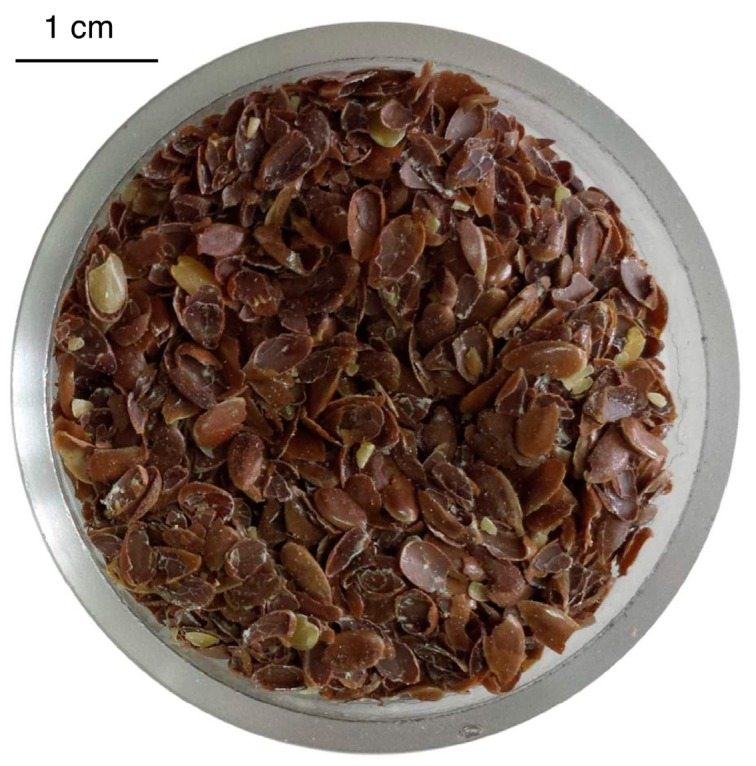
Physical aspect of flaxseed hulls.

Flaxseed (*Linum usitatissimum*) hulls (Figure 1) of the “Comtess” variety were obtained after mechanical dehulling of seeds by abrasion.

### 2.3. Characterization of Flaxseed Hulls

Lipid content was determined by Soxhlet method, as previously reported [30], with slight modification. In brief, 2 g of ground hulls were extracted under reflux with 250 mL petroleum ether for 6 h. The solvent was evaporated using a rotary evaporation system at 50 °C, and oil content was determined by measuring the weight difference before and after extraction.

Dry matter was determined by drying the hulls at 105 °C in an oven until reaching constant weight, according to the “Association Française de Normalisation” (AFNOR) [31]. Total nitrogen content was determined according to Kjeldahl’s method [32], and protein content was determined by multiplying the nitrogen content by 6.25 [5]. Total sugar content was determined as previously described [33]. Total ash was determined after combustion of 5 g hulls for 4 h in a muffle furnace maintained at 550 °C. The mineral composition (K, Ca, Mg, and Mn) of ash was determined by flame atomic absorption spectrometry (Analytic Jena ZEEnit 700 spectrometer, Analytik Jena, Germany) [34].

### 2.4. Soluble Flaxseed Gum Extraction

Soluble flaxseed gum (SFG) were extracted from hulls as previously described [26]. In brief, 20 g of hulls was mixed with 500 mL distilled water in a 1 L rounded bottom flask. The hulls/water mixture was then brought to a boil for 4 h, using heating mantle under reflux. Afterwards, the mixture was recovered and filtered through paper filter under vacuum. The extraction was repeated three times using the same plant material, in order to recover the maximum amount of polysaccharides. The supernatants were pooled and concentrated 20 times using a vacuum rotary evaporation system, maintained at 50 °C. Twenty milliliters of concentrated SFG solution was obtained after this evaporation step. SFG was precipitated overnight by adding 100 mL ethanol 96% (final ethanol concentration = 80%) at −20 °C, and then recovered by 15 min centrifugation at 5000 *g*. The recovered SFG was subjected to five washing steps in order to remove the small molecules. The procedure consists of resuspending the SFG in 20 mL water, precipitation by adding 20 mL of ethanol 96%, and centrifugation as described above. Minerals were removed by dialysis (molecular cutoff = 500 Da) using bidistilled water for 3 days. The obtained desalted samples were freeze-dried and stored at 4 °C until analysis.

### 2.5. Fourier Transform Infrared Spectroscopic Analysis

SFG functional group analysis was assessed by Fourier transform infrared (FTIR) spectroscopy using Analect Instrument fx-6160 (Irvine, CA, USA) as previously described [29]. The transmission (%) was recorded between 650 and 4000 cm^−1^ after mixing 1 mg from the lyophilized SFG sample with 100 mg KBr. The SFG spectrum was then compared to the FTIR spectrum of gum arabic, in order to check for similarities.

### 2.6. Antioxidant Activities of Soluble Flaxseed Gum (SFG)

#### 2.6.1. DPPH Free Radical Scavenging Activity

The ability of SFG to scavenge DPPH free radicals was evaluated as previously described [26]. In brief, different amounts (1–20 mg) of SFG were resuspended in 0.5 mL distilled water, and then mixed with 0.375 mL absolute ethanol and 125 μL DPPH solution (0.02% in ethanol). The mixtures were then vortexed and kept in the dark for 60 min at room temperature. DPPH, initially having deep violet color in solution, becomes colorless or pale yellow in the presence of SFG. Color changes were followed spectrophotometrically (Shimadzu UV/VIS mini 1240, Fisher Scientific, Illkirch, France) at 517 nm, and the absorbance was recorded as A_sample_. A control solution without SFG was prepared under the same conditions by mixing 125 μL DPPH solution with 875 μL absolute ethanol, and the absorbance was recorded as A_control_. A blank solution without DPPH was prepared by mixing SFG (1–20 mg) with 0.5 mL distilled water and 0.5 mL absolute ethanol, and the absorbance was recorded as A_blank_. The free radical scavenging activity (% inhibition) was calculated according to Equation (1).

(1) $$\mathrm{Inhibition}\%=\frac{A_{\mathrm{control}}+A_{\mathrm{blank}}-A_{\mathrm{sample}}}{A_{\mathrm{control}}}\times 100$$

#### 2.6.2. ABTS Free Radical Scavenging Activity

The antiradical activity of SFG was also determined against the free radical 2,2′-azino-bis(3-ethylbenzothiazoline-6-sulphonic acid) (ABTS) based on the discoloration of cations, as previously described [35]. A solution of ABTS (7 µM) was prepared in distilled water and was mixed with a solution of potassium persulfate (2.45 µM). Prior to use, the mixture was kept in the dark for 16 h at room temperature. The resulting intense color matches the ABTS^•+^ radical cations, and the obtained solution was subsequently diluted with distilled water until an absorbance of 0.7 ± 0.02, measured at 734 nm. One milliliter of ABTS^•+^ diluted solution (A_734 nm_ = 0.7 ± 0.02) was mixed with 10 μL of SFG solution at different concentrations (5 to 40 mg·mL^−1^), and the reaction mixture was kept at room temperature for 6 min before measuring the absorbance at 734 nm. The assay relies on the ability of the antioxidant molecules to inhibit the oxidation of ABTS radical cation in ABTS^•+^. SFG scavenging activity (calculated as % of inhibition) was determined according to Equation (2).

(2) $$\mathrm{Inhibition}\%=\left(1-\frac{A}{A_{0}}\right)\times 100$$

where A and A_0_ represent respectively the absorbance values of the solutions containing or not containing the free radical ABTS^•+^.

#### 2.6.3. Total Antioxidant Activity

The total antioxidant activity of SFG was evaluated as previously described [25], with slight modifications. In brief, the experiment consisted of mixing different amounts (5, 7.5, 10, and 15 mg) of SFG with 1 mL of freshly prepared reagent solution (28 mM sodium phosphate, 4 mM ammonium molybdate, and 0.6 M sulfuric acid). The volume in each tube was made up to 1.1 mL with distilled water, and all tubes were kept for 90 min at 90 °C in a thermostatic water bath. Total antioxidant activity was measured at 695 nm after cooling to room temperature and was expressed as ascorbic acid equivalent using a previously established standard curve. A blank solution was prepared by mixing 1 mL reagent with 100 μL distilled water, under the same conditions of the samples.

#### 2.6.4. Reducing Power Capacity

Reducing power capacity of SFG was measured as previously described [36], with slight modifications. Different amounts of SFG (1–20 mg) were mixed with 0.5 mL distilled water, 1.25 mL phosphate buffer (0.2 M, pH 6.6), and 1.25 mL potassium ferricyanide (1% (*w/v*) prepared in water). The mixtures were kept for 20 min at 50 °C in a thermostatic water bath. One milliliter trichloroacetic acid (10% (*w/v*) prepared in water) was added to the reaction mixture after cooling to room temperature. All tubes were then centrifuged for 10 min at 3000 *g*. From each supernatant, 1.5 mL was taken and mixed with 1.5 mL distilled water and 100 μL freshly prepared ferric trichloride solution (0.1% (*w/v*) prepared in water). After vortexing, the absorbance was measured at 700 nm. A blank solution (without SFG) was prepared under the same conditions.

#### 2.6.5. β-carotene Bleaching Test

The test evaluating β-carotene bleaching by SFG was performed as previously described [25], with slight modifications. A fresh mixture solution was prepared by dissolving 0.5 mg β-carotene, 25 μL linoleic acid, and 200 µL Tween 40 in 1 mL chloroform. The solvent used was then evaporated under vacuum at 40 °C, and 100 mL bi-distilled water was added to dissolve the different constituents. Different amounts of SFG (1–20 mg) were mixed with 2.5 mL of the prepared emulsion, and all tubes were kept for 2 h in an oven maintained at 50 °C. β-carotene bleaching was evaluated by measuring the absorbance at 470 nm before and after incubation (A_0h_ and A_2h_, respectively), using Equation (3).

(3) $$\mathrm{Inhibition}\%=(1-\frac{A_{0h\;\left(\mathrm{WSP}\right)}-A_{0h\;\left(\mathrm{control}\right)}}{A_{2h\;\left(\mathrm{WSP}\right)}-A_{2h\;\left(\mathrm{control}\right)}})\times 100$$

where A_0h (control)_ and A_2h (control)_ represent the absorbance values of a control solution (0.5 mL bi-distilled water instead of SFG solution) before and after incubation, respectively.

#### 2.6.6. Statistical Analysis

Antioxidant activities were performed in triplicate, and results were expressed as average values with standard deviation (SD). Multiple sample comparison of the means was done using analysis of variance (ANOVA) (using software SPSS Version 22; IBM^®^ SPSS^®^ Statistics, USA) and was used to determine the significant differences between the results, with a significance level of *p* < 0.05.

## 3. Results and Discussion

### 3.1. Flaxseed Hull Characterization

Table 1 shows the physico-chemical composition of SFG from hulls. Dry matter (7.5 ± 0.7%) was similar to that reported for whole flaxseed, which is in the range of 7%–9%, depending on the variety [37]. Protein content was similar (18.36% ± 0.12%) to whole flaxseed (≈18%) [38], and was slightly higher than that reported for rapeseed hulls (15.27%) [30]. Lipid content was very low (6.53% ± 0.23%, dry basis) compared to that accumulated by whole flaxseed (≈42%) [38]. This content depends on the variety, the moisture content, and the dehulling procedure. It should be noted that oil content in flaxseed hulls was lower than that reported for rapeseed hulls (21.7% ± 1.8%, dry basis), probably due to the easier way to dehull flaxseeds compared to rapeseeds. Total sugars, by contrast to oil content, was relatively high (65.41% ± 1,2%, dry basis), and was higher than that reported for whole flaxseed (28.9%, dry basis) [38]. Total ash (1.99% ± 0.01%, dry basis) was mainly composed of potassium (115.15 ± 0.75 mg/100 g hulls), calcium (115.25 ± 1.35 mg/100 g hulls), and magnesium (52.69 ± 0.53 mg/100 g hulls), with the minor presence of zinc (0.86 ± 0.02 mg/100 g hulls) and manganese (0.48 ± 0.02 mg/100 g hulls).

### 3.2. FTIR Characterization

**Figure 2:**
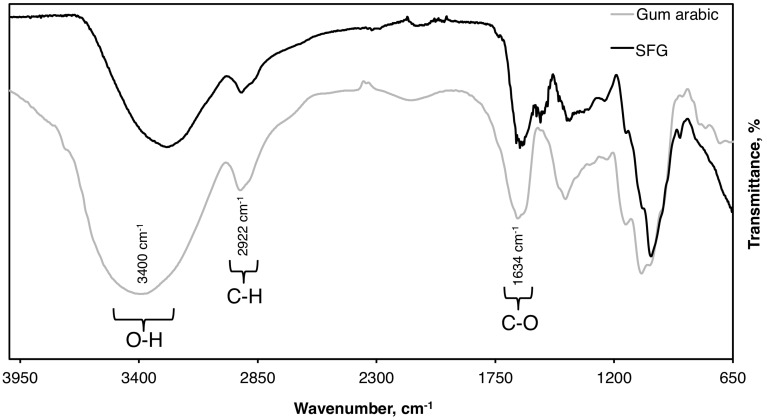
FTIR spectra of soluble flaxseed gum (SFG) and gum arabic. Spectra were recorded between 650 cm^−1^ and 4000 cm^−1^ wavenumber. The most prominent peaks correspond to O-H, C-H, and C-O stretching vibrations.

After lyophilization, SFG presented 12.5% ± 1.19% dry basis, which could represent a considerable amount of polysacchrides to be valorized as food additives. The extracted SFG were characterized by FTIR (650–4000 cm^−1^) and compared to the spectrum of gum arabic (Figure 2). Three characteristic transitions of polysaccharides were observed in the different samples; at 3000–3700 cm^−1^, 1500–1770 cm^−1^, and 950–1200 cm^−1^. Similar results have been reported previously [39,40,41]. Stretching of the hydroxyl groups was confirmed by the presence of the peak at ≈3400 cm^−1^, whereas C-H stretching and bending vibrations are confirmed by the presence of the peak observed at 2922 cm^−1^ [41]. Moreover, the stretching vibrations of C-O bonds are represented by the peak observed at 1634 cm^−1^. It has been previously reported that the presence of pyranose units in the structure is associated with the presence of the bands at 1073 cm^−1^ and 1039 cm^−1^ [41,42,43]. The obtained results demonstrated similar functional groups between SFG and gum arabic, which indicates that SFG probably has a polysaccharidic structure. FTIR data thus provide a preliminary overview on the presence of polysaccharides in the extract. Similar spectra were previously reported for polysaccharides extracted from red prickly pear peels [26], almond, and pistachio juice processing by-products [29], and for almond gum and gum arabic [44,45].

### 3.3. Antioxidant Activity of SFG

**Figure 3:**
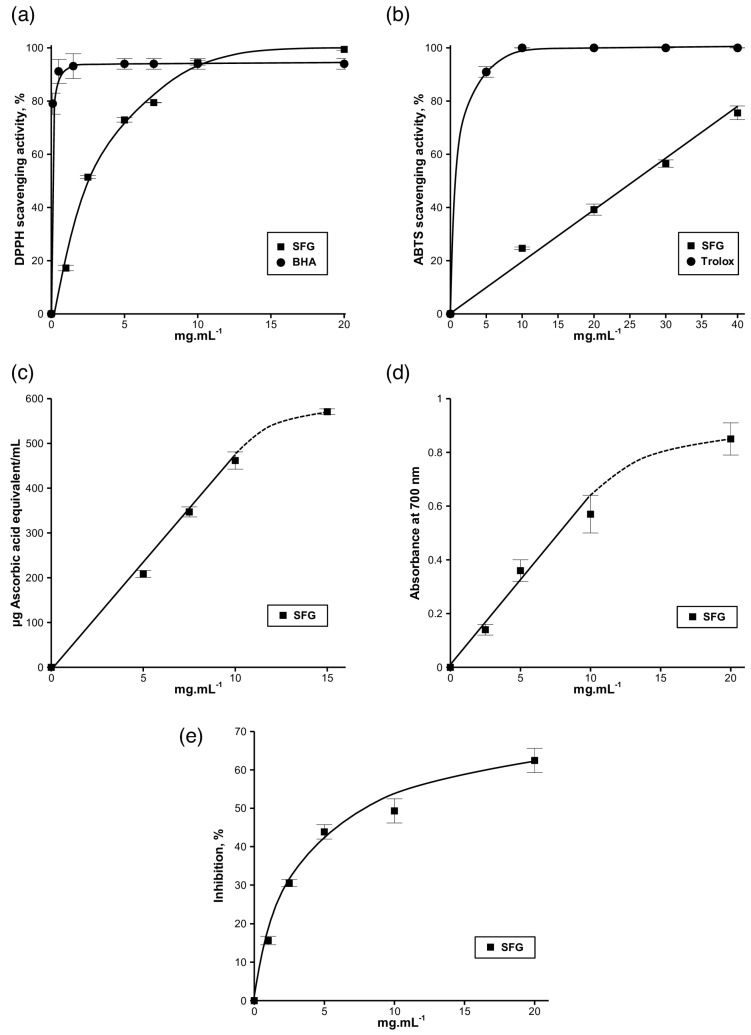
Antioxidant activities of soluble flaxseed gum (SFG): (**a**) 2,2-diphenyl-1-picrylhydrazyl (DPPH) scavenging activity,; (**b**) 2,2′-azino-bis(3-ethylbenzothiazoline-6-sulphonic acid (ABTS) scavenging activity; (**c**) total antioxidant activity; (**d**) reducing power capacity; (**e**) β-carotene bleaching activity. Trolox and BHA were used as standard molecules. Continuous lines are given to guide the eye.

Antioxidant molecules are able to reduce the stable radical DPPH, having a deep violet color in solution, to the yellow-colored 1,1-diphenyl-2-picrylhydrazyl. The free radical scavenging activity of SFG was assessed by DPPH test (Figure 3a). The obtained results showed that the percentage of inhibition was proportional to the concentration of SFG used. The highest DPPH free radical scavenging activity (≈100%) was observed beyond ≈10 mg·mL^−1^. The half inhibition concentration (IC_50_) value of SFG was also determined, corresponding to 2.5 mg·mL^−1^. The lowest IC_50_ value corresponds to the highest DPPH scavenging activity. Results demonstrated that the IC_50_ value of SFG was lower than that reported for other polysaccharides, such as those extracted from guara fruits (IC_50_ = 10.8 mg·mL^−1^) [43], red prickly pear peels (IC_50_ = 10.8 mg·mL^−1^) [26], and almond juice processing by-products (IC_50_ = 2.87 mg·mL^−1^) [29], and was higher than that found for polysaccharides extracted from garlic straw (IC_50_ = 740 μg·mL^−1^) [28] and pistachio juice processing by-products (IC_50_ = 1.61 mg·mL^−1^) [29]. Compared to BHA, SFG showed good scavenging activity, which demonstrates its ability to react with free radicals and reduce them to more stable products.

Similarly to DPPH free radical, ABTS showed potential scavenging activity and again demonstrated its ability to reduce free radicals and convert them to more stable products. Results are presented in Figure 3b and show that the scavenging activity is proportional to the concentration of SFG. The highest percentage of inhibition (75.6% ± 2.6%) was observed at 40 mg·mL^−1^, which was lower than that observed for DPPH at the same concentration. The IC_50_ value of SFG was also determined, corresponding to 26.1 mg·mL^−1^. However, ABTS scavenging activity remains lower than that observed for Trolox, used as standard.

The reduction of phosphomolybdate was followed by the measurement of total antioxidant capacity (TAC). A green complex of phosphate/Mo (V) is formed at acidic pH, which has a maximal absorbance at 695 nm. The TAC of SFG extracted from hulls was determined as described previously, and was expressed as ascorbic acid equivalent (AAE)/mL (Figure 3c). The obtained results indicate that the TAC was proportional to the concentration of SFG, with linearity up to 10 mg·mL^−1^. At this concentration, 10 mg of SFG was equivalent to 461 mg ascorbic acid. Beyond this concentration, TAC continues to increase with lower slope, indicating saturation of the medium. Although the lower antioxidant activity compared to that of ascorbic acid, the obtained results indicate that SFG represents an interesting natural antioxidant molecule.

Moreover, the reducing capacity of SFG to convert Fe^3+^ to Fe^2+^ was followed by measuring the absorbance (A) at 700 nm, corresponding to the formation of Perl’s Prussian blue at 700 nm (Figure 3d). The obtained results indicate that SFG reducing power was proportional to the concentration of SFG. The absorbance showed linearity until 10 mg·mL^−1^, similar to TAC, and then the slope decreased beyond this concentration. Although the results were promising, SFG reducing capacity was lower than that reported in the literature for other polysaccharides, such as those extracted from almond juice processing by-product (A_700 nm_ = 1.5 at 5 mg·mL^−1^) [41], pistachio juice processing by-product (A_700 nm_ = 1.74 at 5 mg·mL^−1^) [41], mushroom (A_700 nm_ = 3.4 at 20 mg·mL^−1^) [46], and from almond gum (A_700 nm_ = 1.36 at 12 mg·mL^−1^) [27].

Finally, the β-carotene bleaching test simulates the oxidation of lipids’ membrane; thus, it is considered a good model for membrane-based lipid peroxidation. The oxidation of linoleic acid during this assay generates peroxyl free radicals, which are able to oxidize the unsaturated β-carotene. The presence of an antioxidant molecule in the medium will minimize this oxidation. Therefore, the antioxidant activity of SFG could be measured by following the degradation rate of β-carotene. The obtained results (Figure 3d) show that the inhibition increased proportionally to the concentration of SFG up to 20 mg·mL^−1^. The maximum measured inhibition corresponding to this concentration was 62.4% ± 3.1%, thus showing the efficiency of SFG as a natural antioxidant.

## 4. Conclusions

In conclusion, the in vitro antioxidant capacity of soluble flaxseed gum extracted from hulls was demonstrated. Results showed great antioxidant activities through numerous tests, and were compared to commercial standard molecules. SFG presented similar functional groups when compared to gum arabic, which suggests its polysaccharidic structure. These results show the promising potential of flaxseed hulls as a co-product to be fully exploited and extract food additives that could replace other commercial gums, such as gum arabic. However, further studies should be conducted to explain the mechanisms behind these activities.

## Figures and Tables

**Table 1:** Physico-chemical composition of flaxseed hulls.

Compound	Dry Basis %	Dry Matter mg/100 g
Dry matter	7.5 ± 0.7	
Proteins	18.36 ± 0.12	
Lipids	6.53 ± 0.23	
Sugars	65.41 ± 1,2	
Ash	1.99 ± 0.01	
K^+^		115.15 ± 0.75
Ca^2+^		115.25 ± 1.35
Mg^2+^		52.69 ± 0.53
Zn^2+^		0.86 ± 0.02
Mn^2+^		0.48 ± 0.02
